# New Considerations for Collecting Biomechanical Data Using Wearable Sensors: How Does Inclination Influence the Number of Runs Needed to Determine a Stable Running Gait Pattern?

**DOI:** 10.3390/s19112516

**Published:** 2019-06-01

**Authors:** Nizam U. Ahamed, Lauren C. Benson, Christian A. Clermont, Andrew J. Pohl, Reed Ferber

**Affiliations:** 1 Faculty of Kinesiology, University of Calgary, Calgary, AB T2N 1N4, Canada; nizam.ahamed1@ucalgary.ca (N.U.A.); lauren.benson@ucalgary.ca (L.C.B.); christian.clermont@ucalgary.ca (C.A.C.); andrew.pohl@ucalgary.ca (A.J.P.); 2 Faculty of Nursing and Cumming School of Medicine, University of Calgary, Calgary, AB T2N 1N4, Canada; 3 Running Injury Clinic, University of Calgary, Calgary, AB T2N 1N4, Canada

**Keywords:** accelerometer, gait, elevation, inertial measurement unit, running, slope

## Abstract

As inertial measurement units (IMUs) are used to capture gait data in real-world environments, guidelines are required in order to determine a ‘typical’ or ‘stable’ gait pattern across multiple days of data collection. Since uphill and downhill running can greatly affect the biomechanics of running gait, this study sought to determine the number of runs needed to establish a stable running pattern during level, downhill, and uphill conditions for both univariate and multivariate analyses of running biomechanical data collected using a single wearable IMU device. Pelvic drop, ground contact time, braking, vertical oscillation, pelvic rotation, and cadence, were recorded from thirty-five recreational runners running in three elevation conditions: level, downhill, and uphill. Univariate and multivariate normal distributions were estimated from differing numbers of runs and stability was defined when the addition of a new run resulted in less than a 5% change in the 2.5 and 97.5 quantiles of the 95% probability density function for each individual runner. This stability point was determined separately for each runner and each IMU variable (univariate and multivariate). The results showed that 2–4 runs were needed to define a stable running pattern for univariate, and 4–5 days were necessary for multivariate analysis across all inclination conditions. Pearson’s correlation coefficients were calculated to cross-validate differing elevation conditions and showed excellent correlations (r = 0.98 to 1.0) comparing the training and testing data within the same elevation condition and good to very good correlations (r = 0.63–0.88) when comparing training and testing data from differing elevation conditions. These results suggest that future research involving wearable technology should collect multiple days of data in order to build reliable and accurate representations of an individual’s stable gait pattern.

## 1. Introduction

As the gait biomechanics research community begins to use inertial measurement units (IMUs) to capture gait data in real-world environments [1,2,3,4,5], guidelines for the use of IMUs during continuous monitoring and multi-day data collections of running gait are required [4]. For example, traditional biomechanics research, involving laboratory-based data collections, generally involve well-controlled external factors, such as temperature and running elevation and only collect a limited number of strides and/or trials [1,4,5]. Considering that changing external factors, such as fluctuations in ambient temperature [2] and even whether the activity took place on a weekday or weekend [6] can affect real-world data collections using IMUs, it is important to quantify the number of sessions needed to define an individual’s ‘typical’ activity pattern.

Until recently it has been suggested that “a few days” of data are necessary in order to establish a “relatively stable” gait pattern [7], however, no specific information or guidelines were provided. In contrast, a recent study by Benson et al. [5] reported that 4–5 days of data are necessary in order to determine a typical gait pattern across multiple days of data collection. However, three significant limitations were apparent in this study that need to be addressed. First, these authors [5] only analysed data from 12 recreational runners, making it difficult to generalise their results to larger populations of runners [5]. Second, data were only collected from runs that were limited to 4–5 km in total distance and only 2.5 km of accumulated data from each run were analysed. Third, and most importantly, these authors constrained their analysis to only level sections of outdoor running and did not consider how running uphill or downhill would influence the ability to establish a stable gait pattern.

It has been shown that uphill and downhill running can greatly affect gait biomechanics [8,9,10]. For example, Vernillo et al. [10] reported that the increased demands for work as running slope increases are accompanied by an increase in power output at all joints, particularly the hip, along with an overall progressive move from a rearfoot strike towards a mid- to forefoot strike pattern as running incline increases. In contrast, they reported that the rearfoot strike pattern associated with downhill running was related to increased braking forces. Additionally, it has been reported that uphill or downhill running has several fatigue-related intrinsic features compared with level running that could also affect gait biomechanics, especially over longer distances [8,11]. For example, Kowalski and Li [11] reported that when compared to level running, braking forces were larger during downhill conditions and propulsive forces were larger during uphill conditions. Additionally, Ahamed et al. [3] reported that braking force was the most important variable for discriminating between uphill and downhill running for a group of recreational runners. Therefore, considering that uphill and downhill running demand significant alterations in gait biomechanics, and considering that uphill and downhill running are commonly experienced by runners in real-world conditions, it remains unknown how many individual runs are required to establish a stable gait pattern during uphill and downhill running compared to that previously reported for level running.

The purpose of this study was to determine the number of runs needed to establish a stable running pattern during level, downhill, uphill conditions as well as for an entire run irrespective of elevation (mixed condition). To assess this hypothesis, both the univariate and multivariate analyses of running biomechanical data were collected using a single IMU device. We hypothesized that 4–5 runs would be needed to determine a stable gait patterns for each of the three elevation conditions but that more runs would be necessary for the mixed condition.

## 2. Materials and Methods

### 2.1. Participants

Based on an a priori power analysis (α = 0.05, β = 0.20), 35 recreational runners (25 females: age = 47.6 ± 11.1 years; height = 165.1 ± 5.2 cm; mass = 64.7 ± 8.2 kg; and 10 males: age = 54.8 ± 9.6 years; height = 174.9 ± 5.8 cm; mass = 80.5 ± 7.8 kg) volunteered to participate in this study. The runners were free of any neuromuscular diseases or musculoskeletal injuries and were registered for a marathon training program managed by a local running group. This protocol was approved by the University of Calgary Conjoint Health Research Ethics Board (REB16-2035), and all the runners provided their written informed consent.

### 2.2. Instrumentation

Biomechanical gait variables from each runner were recorded using a commercially available wearable IMU (Lumo Run^®^; Lumo Bodytech Inc., Mountain View, CA, USA; sampling rate of 100 Hz and recording data every five strides), consisting of a three-dimensional accelerometer, magnetometer, and gyroscope. According to the manufacturer’s instructions, the IMU was attached to the back of the shorts or to a running belt near the individual’s center of mass. During each run, the IMU performed on-board computation of six biomechanical variables: pelvic drop (deg), vertical oscillation (cm), ground contact time (ms), braking (m/s), pelvic rotation (deg) and cadence (steps/min). A GPS watch (Garmin vívoactive^®^ HR; Garmin International Inc., Olathe, KS, USA) was attached to each runner’s preferred wrist and recorded data (1 Hz) on the runner’s speed (m/s), distance (km), and global positioning data, including latitude, longitude and altitude (Supplementary S2). For each run, the runner’s clothes, shoes, device placement, pace and running route were not controlled. A MATLAB-based (MATLAB^®^ 2017a) program was developed to synchronize the IMU and GPS data for further analysis.

### 2.3. Data Collection

Runners wore both the IMU and the GPS watch during all training runs over the course of their marathon training program. Seven pain-free runs, of at least 12 km in length, were performed by each runner and used in the analysis. For each runner, no more than 1 run was recorded on a single day with an average of 12.9 days (± 6.54) between the collective runs. Data from each individual run were considered based on the following four conditions; (i) mixed condition: (the entire run and irrespective of elevation), (ii) level: (±2%), (iii) uphill: (+3% to +15%), and (iv) downhill: (−3% to −15%) running conditions.

To minimize the potential effects of fatigue, only data from the first 10 km of each run were used with data from the first kilometer discarded and considered as a warmup period. Each run was segmented into elevation sections of at least 100 m in length. A 10 s moving average was used to smooth elevation and speed data from the GPS watch and sections where running speed was less than 1.8 m/s [12] were removed so that only data where subjects were actively running was used in the analysis. The average elevation gain or loss was computed for continuous non-overlapping windows of 100 m length, which were then segmented into each terrain type. A summary of these data is provided in Table 1.

### 2.4. Data Analysis

A detailed description of the data analysis process is described in detail elsewhere [5]. In brief, to determine the number of runs needed to establish a stable running pattern, training datasets consisting of varying numbers of runs were created, and the runs that did not form part of a training dataset comprised the corresponding testing datasets. A leave-one-out cross-validation approach was used to determine the level of similarity between all training sets from the thirty-five runners for each of the four conditions and a test run performed in the same condition. Thus, 7-N elevation-specific testing datasets were obtained for each unique training dataset, where N is the number of runs in the training dataset (Table 2). For each unique training dataset, the 95% probability density function of the univariate or multivariate normal distribution was determined using the multivariate normal probability density function (MVNPDF) (Supplementary S1) function in MATLAB [13,14], and these analyses were performed for each of the IMU variables individually (univariate analyses) and for all of the IMU variables together (multivariate analyses) (Supplementary S3).

A threshold of the resulting 95% probability density function, epsilon, was determined such that 95% of the training data had a probability greater than or equal to epsilon. The data points in the testing dataset with a probability greater than or equal to epsilon were considered similar to the training dataset, and the percentage of similar data points in the testing dataset was recorded. Univariate and multivariate normal distributions were estimated from differing numbers of runs and stability was defined when the addition of a new run resulted in less than a 5% change in the 2.5 and 97.5 quantiles of the estimated density for each individual runner. This stability point was determined separately for each runner and each IMU variable (univariate and multivariate). The overall stability point for each IMU variable was determined as the maximum number of runs needed to reach stability among the thirty-five runners for each of the four elevation conditions.

In order to further cross-validate our approach, a crossover condition analysis was done by randomly selecting five runs for each individual as training runs, with the remaining two runs designated as test runs. For each condition, a model was trained using data from the training runs only, and data in each condition from the testing runs was used as separate test sets (e.g., train on uphill from five training runs, separate tests with uphill, downhill, level, and all from two testing runs). The number of data points outside the 95% probability density function of the trained model was the outcome variable. This procedure was repeated so that a model was trained for each of the four conditions, and for all variables. The association between the number of data points outside the 95% probability density function for each train/test combination in the crossover condition analysis and the number of data points outside the 95% probability density function at the five-run stability point in the original analysis was determined using Pearson’s correlation coefficient. Based on previous studies, we defined r < 0.60 as poor, 0.61–0.80 as good, 0.81–0.95 as very good, and >0.95 as excellent [15,16]. All statistical tests were conducted using Minitab^®^ statistical software (Minitab Inc., State College, PA, USA).

## 3. Results

The average and maximum number of runs needed to define stable running patterns for each elevation condition, and for the data from the entire run, are shown in Table 3. Overall, the results showed that 2–4 days were needed to define a stable running pattern for univariate and 4–5 days were necessary for multivariate analysis. Cadence required the fewest number of days to reach stability especially during downhill running (two days) compared to level and uphill running (three days) and also compared to the other univariate measures (3–4 days). The multivariate analysis required 4–5 days to reach stability irrespective of elevation condition.

The cross-validation based on the Pearson’s correlation coefficient and comparison of the average number of data points that fell outside of the 95% probability density function showed excellent to perfect correlations (r = 0.98 to 1.0) comparing the training and testing data within the same elevation condition (Table 4). However, if the training and testing data were from different elevation conditions, the correlations were considered to be between good and very good (r = 0.63 to 0.88) for level, uphill, and downhill conditions and also good to very good for the mixed condition (r = 0.68 to 0.92).

## 4. Discussion

The purpose of the current study was to determine the number of runs needed to establish a stable or typical running pattern during real-world running conditions of level, uphill, and downhill running grade based on both univariate and multivariate analyses. In partial support of our hypotheses, the first major finding of the current study was, despite a sample almost three times as large as Benson et al. [5], 3–4 runs were still necessary to define a stable running pattern when utilising univariate and five runs were necessary for a multivariate analysis. These findings are also similar to Aguilar–Farias et al. [6] who also suggested that five days were needed to reliably estimate weekly activities such as time spent walking, sitting, lying down, and standing in a large group of healthy adults using a wearable activity sensor. Thus, the current study provides further evidence that future research involving wearable technology, during real-world conditions should collect at least 3–5 days of data, depending on the statistical approach and variable of interest, in order to build reliable and accurate representations of the population of interest.

Another novel finding of this study is that uphill and downhill running conditions required a different number of data collections, when compared to level running (i.e., uphill vs. level and downhill vs. level), for both multivariate (four days vs. five days) and univariate (2–4 days vs. 3–4 days) methods of analysis to reach stability. Reasons for these findings are speculative but could be due to a more constrained, or predictable running gait pattern being exhibited by the runners for uphill and downhill running as compared to level running.

As well, when considering the running data as a whole (mixed condition), 3–4 days of data were needed for univariate and five days were needed for multivariate analysis. Again, these results are most likely due to greater variability of gait biomechanical characteristics demonstrated within the mixed condition, as compared to any single grade conditions. Therefore, variance of univariate and multivariate distributions would be larger for the same number of runs, as compared to a single elevation condition.

The braking and cadence variables exhibited a different number of data collections, dependent on the grade, to reach stability. The braking variable required more data (four days) to establish a stable gait pattern during uphill and downhill running compared to level running (three days). One possible reason is that this biomechanical variable was more inconsistent, or unpredictable, during uphill and downhill running compared to level running and therefore resulted in a greater variance as compared to level running. In contrast, cadence only required two days of data during downhill running as compared to three days of data for the other running conditions suggesting a more predictable/consistent gait pattern during the downhill condition.

While the cross-validation approach used by Benson et al. [5] involved an additional group of testing datasets being generated using the runs by all other runners, we thought it is important to utilise a different method to cross-validate our findings. Overall, we found that compared to the near-perfect correlations for similar training and testing datasets (e.g., uphill vs. uphill), the correlations were only good to very good comparing dissimilar training and testing datasets (e.g., uphill vs. downhill). These results provide an additional level of confidence for the overall approach of building training sets based on unique sets of extrinsic factors (e.g., degree of inclination). However, the multivariate analysis of the mixed model used in the current study required five days to reach stability, which was similar to the level model, and only required one additional run to reach stability as compared to the multivariate uphill and downhill models. Thus, we suggest that separate models for different running inclinations conditions are not needed when utilising a multivariate statistical approach to data analysis. As well, these results were generally similar for the six univariate models suggesting that future research does not necessarily need to segment out specific elevation conditions for data collected during real-world runs in order to calculate stability in gait patterns. However, limitations in the current study demand further research to better understand the complex association between intrinsic and extrinsic factors and how gait patterns are affected.

Limitations to this study are acknowledged. First, the current study used only a single IMU as it was designed to build upon the research conducted by Benson et al. [5] and include more individuals and different running elevations. We acknowledge that additional IMU sensors, placed in different locations of the body, may yield different results. Regardless, it is important to consider that the current study involved collecting data from runners over multiple days and in real-world environments. Thus, we had to strike a balance between a runner’s comfort and what they would tolerate in terms of attached devices. The IMU used in the current study was also a commercially-available wearable device with non-transparent data processing procedures. Therefore, future research should include a broader range of biomechanical variables and also involve sensors placed on multiple locations of the body. Secondly, the runners had no limitations on running route or clothing and/or footwear options, each of which could influence running gait patterns. Thus, future research should consider these factors as well as other intrinsic and extrinsic factors such as running surfaces, fatigue levels [17,18], state of physical activity such as sleep, running speed and heart rate [19,20,21,22], and ambient temperatures [23]. As well, we acknowledge that different outdoor conditions, such as running on an outdoor track versus the outdoor paved run path used in the present study, may yield different results. Third, it is important to note that the current study and that of Benson et al. [5] represent one possible way to define the number of runs needed to establish a stable running pattern. As biomechanical research moves outside of the traditional laboratory setting, we recommend researchers continue to develop similar novel and objective methodological approaches to better define subject-specific models of gait patterns in order to build reliable and accurate representations of the population of interest. This premise is further supported by Ahamed et al. [3] who reported that subject-specific models can better characterize changes in gait biomechanical patterns during level, uphill, and downhill elevation conditions compared to a more traditional group-based approach. We also acknowledge that the placement of the sensor was not strictly controlled and may have influenced the results. Specifically, the current study involved an IMU being clipped to the posterior aspect of either the runner’s waistband or running belt, and there was a possibility that the IMU could shift slightly during each of the seven runs. However, there was an equal chance of the IMU shifting position for any participant and at any time, regardless of whether they were running uphill, downhill, or on level ground and regardless of whether they changed from attaching the IMU to their waistband or a running belt, from one run to the next. Therefore, we do not feel that the results of the study could be significantly influenced by such random and minor occurrences, but future research may want to account for this factor. Finally, the current study was based on a sample of convenience and involved 25 female and 10 male runners who involved in the same marathon training program. It is well known that male and female runners exhibit different gait biomechanical patterns [24,25] and that female runners have a two-fold risk of sustaining certain running-related injuries as compared to their male counterparts [26]. However, these studies [24,25] were based on laboratory testing conditions and very few studies have investigated whether these sex-based differences exist in real-world settings [27]. Therefore, future research is necessary.

Despite these limitations, we hope that these results help clinicians understand and prevent running-related injuries and also help coaches improve the athletic performance of runners. For example, it is possible for a clinician to gauge an injured athlete’s rehabilitation progress by altering and/or gradually changing any one of the univariate biomechanical measures [28] and comparing these changes to a baseline stable measure until they fall within 5% of the athlete’s pre-injury probability density function. As well, the relationship between typical and non-typical running may be related to injury risk and this should be a focus of future work. For example, continuous monitoring of running gait could play a role in determining the repetitive loading cycles at stresses below the stress limit of a material, a process referred to as mechanical fatigue by Edwards et al. [29]. These authors [29] put forth a theoretical foundation and operational framework necessary to model overuse injury as a mechanical fatigue phenomenon but also suggested that 1000 s of repetitive cycles were necessary for more accurate measures of tissue loading. By using wearable sensors, we hope that theoretical models, such as those put forth by Edwards et al. [29], can begin to investigate the potential interaction between loading magnitude (e.g., activity, intensity) and activity exposure (e.g., volume, mileage) in real-world settings in order to reduce the health burden of running-related injuries.

In conclusion, the current study showed that 2–4 runs were needed to define a stable running pattern for univariate and 4–5 days were necessary for multivariate analysis using data from a single IMU during real-world running conditions of level, uphill, and downhill running conditions. These results play an important role in biomechanical gait research using wearable sensors outside the laboratory setting as they provide an objective methodological approach in order to build reliable and accurate models of gait biomechanical patterns in real-world environments. These results also provide a foundation for future biomechanical research and clinical practice that can define a stable running pattern in order to identify atypical biomechanical gait patterns, aid clinicians to rehabilitate running-related injuries, and help coaches improve the athletic performance of runners.

## Figures and Tables

**Table 1:** Means and standard deviations of the characteristics for each of the four running conditions.

Elevation Condition	Total Distance (km)		Average Inclination (%)		Speed (m/s)	
	Mean	SD	Mean	SD	Mean	SD
Level	4.2	2.1	1.88	1.44	2.41	0.26
Uphill	3.3	1.1	3.24	1.61	2.43	0.25
Downhill	3.5	1.3	−3.81	2.52	2.44	0.22
Mixed	8.3	3.2	0.17	2.51	2.45	0.25

**Table 2:** Unique sets of runs based on number of runs (train-test pairs) included in each set.

Number of Runs Per Set	Number of Unique Sets	Sets	Number of Training-Testing Dataset Pairs
1	7	{Run 1}, {Run 2}, …	49
2	21	{Runs 1 + 2}, {Runs 1 + 3}, …	126
3	35	{Runs 1 + 2 + 3}, {Runs 1 + 2 + 4}, …	175
4	35	{Runs 1 + 2 + 3 + 4}, {Runs 1 + 2 + 3 + 5}, …	140
5	21	{Runs 1 + 2 + 3 + 4 + 5}, {Runs 1 + 2 + 3 + 4 + 6}, …	63
6	7	{Runs 1 + 2 + 3 + 4 + 5 + 6}, {Runs 1 + 2 + 3 + 4 + 5 + 7},	14
7	1	{Runs 1 + 2 + 3 + 4 + 5 + 6 + 7}	0

**Table 3:** Number of days (mean, standard deviation (SD) and maximum) needed to define a stable running pattern (univariate and multivariate).

Units	Variable/Condition	Cadence	Bounce	Braking	Pelvic Drop	Pelvic Rotation	Ground Contact Time	Multivariate
Mean (SD)	Level	1.71 (0.67)	2.09 (0.61)	2.46 (0.61)	2.60 (0.81)	2.26 (0.71)	2.34 (0.76)	3.29 (0.79)
	Uphill	1.74 (0.56)	2.10 (0.66)	2.61 (0.85)	2.49 (0.82)	2.11 (0.68)	2.17 (0.75)	3.14 (0.65)
	Downhill	1.37 (0.49)	2.11 (0.63)	2.46 (0.78)	2.40 (0.74)	1.97 (0.66)	2.29 (0.57)	3.20 (0.53)
	Mixed	1.69 (0.63)	2.17 (0.82)	2.26 (0.56)	2.63 (0.73)	2.27 (0.71)	2.23 (0.59)	3.21 (0.72)
Max	Level	3	3	3	4	4	4	5
	Uphill	3	3	4	4	3	3	4
	Downhill	2	3	4	4	3	3	4
	Mixed	3	4	3	4	4	3	5

**Table 4:** Correlation matrices of similar data points between training and testing data sets. Grey cells indicate resultant r-values when training and testing data sets were from the same running conditions

Variables		Test	Level	Uphill	Downhill	Mixed
	Train					
Cadence	Level		0.98	0.75	0.76	0.88
	Uphill		0.77	1.0	0.67	0.86
	Downhill		0.67	0.66	1.0	0.82
	Mixed		0.82	0.85	0.85	0.99
Vertical Oscillation	Level		0.99	0.76	0.69	0.92
	Uphill		0.74	0.99	0.71	0.91
	Downhill		0.63	0.73	1.0	0.85
	Mixed		0.81	0.82	0.68	1.0
Braking	Level		1.0	0.66	0.73	0.84
	Uphill		0.78	1.0	0.76	0.86
	Downhill		0.71	0.74	0.99	0.84
	Mixed		0.85	0.84	0.81	1.0
Pelvic Drop	Level		0.99	0.68	0.72	0.81
	Uphill		0.75	0.98	0.68	0.83
	Downhill		0.76	0.75	1.0	0.84
	Mixed		0.83	0.81	0.65	1.0
Pelvic Rotation	Level		1.0	0.76	0.69	0.84
	Uphill		0.72	0.99	0.67	0.87
	Downhill		0.73	0.72	0.99	0.86
	Mixed		0.81	0.85	0.86	1.0
Ground Contact Time	Level		0.99	0.72	0.66	0.70
	Uphill		0.74	1.0	0.68	0.89
	Downhill		0.71	0.71	1.0	0.82
	Mixed		0.84	0.83	0.88	1.0
Multivariate	Level		0.98	0.63	0.74	0.73
	Uphill		0.62	1.0	0.76	0.66
	Downhill		0.70	0.73	0.99	0.68
	Mixed		0.67	0.71	0.69	1.0

## Data Availability

The data generated and/or analyses for to the current study are available from the corresponding author upon reasonable request.

## Supplementary Materials

The following are available online at https://www.mdpi.com/1424-8220/19/11/2516/s1: Sample Matlab Code_Anomaly Detection; S2: Sample Data from Garmin Wearable; S3: Sample Data from Lumo Wearable.
