# Supplementary material for: New Considerations for Collecting Biomechanical Data Using Wearable Sensors: How Does Inclination Influence the Number of Runs Needed to Determine a Stable Running Gait Pattern?
Source: Sensors (Basel). 2019 Jun 1;19(11):2516. doi: 10.3390/s19112516 (PMC6603692; doi:10.3390/s19112516)
Supplement: Supplementary file 1 [file sensors-19-02516-s001.zip › Suppl/Sample Matlab COde_Anomaly Detection.pdf]

```matlab
% Script Writers: Running Injury Clinic Team, University of Calgary, Canada
%Date: 2019/02/01
% Copyright: Running Injury Clinic, University of Calgary


%% Anomaly Detection Algorithm

[m,n] = size(train);
[mm,nn] = size(test);


%  Estimate mu and sigma2 from train
mu = zeros(n, 1);
sigma2 = zeros(n, 1);

for i = 1:n
    mu(i) = mean(train(:,i));
    sigma2(i) = var(train(:,i));
end

%  Train algorithm

k = length(mu);

if (size(sigma2, 2) == 1) || (size(sigma2, 1) == 1)
    sigma2 = diag(sigma2);
end

train = bsxfun(@minus, train, mu(:)');
p = (2 * pi) ^ (- k / 2) * det(sigma2) ^ (-0.5) * ...
    exp(-0.5 * sum(bsxfun(@times, train * pinv(sigma2), train), 2));

% Detemine epsilon based on 5% of training data
[min,index]=sort(p);
epsilon = min(round(0.05*m));

%  Determine p for new run
pval = multivariateGaussian(test, mu, sigma2);

% Predict anomalies in test set based on data from train
%Anomaly = 1, Normal = 0
for i = 1:mm
    predictions(i) = (pval(i) < epsilon);
end

% Calculate the total number of anomalies in test set
anomalies = sum(predictions == 1);

percent_similar = (mm-anomalies)/mm*100;

end
```
